# The Association of Simultaneous Increase in Interleukin-6, C Reactive Protein, and Matrix Metalloproteinase-9 Serum Levels with Increasing Stages of Colorectal Cancer

**DOI:** 10.1155/2018/2830503

**Published:** 2018-07-30

**Authors:** Ismar Rasic, Velma Rebic, Azra Rasic, Goran Aksamija, Svjetlana Radovic

**Affiliations:** ^1^Clinic for General and Abdominal Surgery, Clinical Center of the University of Sarajevo, Sarajevo, Bosnia and Herzegovina; ^2^Department of Microbiology, Faculty of Medicine, University of Sarajevo, Sarajevo, Bosnia and Herzegovina; ^3^Clinic for Oncology, Clinical Center of the University of Sarajevo, Sarajevo, Bosnia and Herzegovina; ^4^Department of Pathology, Faculty of Medicine, University of Sarajevo, Sarajevo, Bosnia and Herzegovina

## Abstract

**Background:**

Tumor development and growth are driven in many cases by inflammatory cells, which can produce cytokines and other factors that can stimulate the development of the malignant process. The aim of this study was to evaluate interleukin-6 (IL-6), C-reactive protein (CRP), matrix metalloproteinase-9 (MMP-9), serum levels in patients with colorectal cancer (CRC), and their association with the stage of CRC.

**Methods:**

IL-6, MMP-9, and CRP serum levels were measured in 75 patients with CRC just before surgical treatment, as well as in 20 healthy individuals as controls. Surgically obtained tissue material was subjected to pathological analysis.

**Results:**

Significant increase in CRP and IL-6 serum concentration is associated with increasing stage of CRC (*p* <0.05), where MMP-9 serum level was significantly higher in stages III and IV compared to the stage II CRC. Significant correlation was found between IL-6 and MMP-9 serum levels (rho=0.478;* p* <0.001) as well as between IL-6 and CRP serum levels (rho=0.720;* p* <0.001) and between MMP-9 and CRP serum levels (rho=0.379;* p* <0.001). Serum levels of MMP-9 and CRP have been shown to be independent predictors of the CRC stage.

**Conclusion:**

Combined quantification of IL-6, MMP-9, and CRP serum levels seems to be a reliable index of inflammation-related processes during colorectal carcinogenesis.

## 1. Introduction

Colorectal cancer (CRC) is one of the most frequent causes of malignant morbidity worldwide and the second most frequent cause for cancer-related deaths in Europe [1, 2].

The role of inflammation in the initiation and progression of colorectal cancer has been the subject of intensive research in recent years. Patients with inflammatory bowel disease (IBD) such as Crohn's disease (CD) and ulcerative colitis (UC) have an increased risk for the development of CRC. Colorectal cancer is observed in 5.5-13.5% of all patients with ulcerative colitis and 0.4-0.8% of patients with Crohn's disease [3]. According to meta-analysis Eaden et al., cumulative risk for CRC is 1.6% at 10 years, 8.3% at 20 years, and 18.4% at 30 years of UC duration [4].

In epidemiological studies, chronic intestinal inflammation was closely associated with the risk of CRC, and several proinflammatory cytokines released from immune and other cells infiltrated into the microenvironment are suggested to regulate tumor initiation and progression [5]. In recent years the pleiotropic cytokine interleukin-6 (IL-6) and its intracellular signaling pathways, mostly JAK1/2-Stat3, attracted the attention of researchers as a possible link that connecting chronic inflammation and CRC promotion, although the mechanisms of its activation and the contribution to the pathogenesis of chronic inflammatory diseases and cancer are not fully understood [6–8]. Some studies have shown elevated serum and cancer tissue IL-6 levels in CRC patients, and its concentration is correlated with tumor size, metastasis, and reduced survival [9–11].

Inflammatory cytokines are potent activators of matrix metalloproteinases (MMPs) which belong to a family of endopeptidases with proteolytic activity. These enzymes are capable of degrading all kinds of extracellular matrix proteins including tumor matrix proteins, allowing tumor cells to invade the surrounding connective tissue, enter and exit the blood vessels, and metastasize to distant organs [9]. Matrix metalloproteinase-9 (MMP-9) is an important member of the matrix metalloproteinase family. Many of the studies conducted up to date on the MMP-9 in CRC are focused on the correlation of its expression in tissue and the clinicopathological features of the tumor [12, 13]. Recent publications about colorectal cancer indicate that matrix metalloproteinase-9 (MMP-9) serum levels are elevated in the patients with colorectal cancer [14, 15]. Increased MMP-9 serum concentration is of special interest in the progression of this cancer and might also play a role in the carcinogenesis from adenoma to colorectal carcinoma [16]. Although numerous studies have greatly improved the knowledge of colorectal tumor genesis, the association between parameters of chronic inflammation such as IL-6, C reactive protein (CRP) and MMP-9 is not fully understood as well as their relation to the progression of CRC.

The aim of this study to evaluate mutual relation between IL-6, CRP and MMP-9 serum levels in patients with CRC and their association with staging and clinical-pathological features of colorectal cancer. 

## 2. Material and Methods

### 2.1. Patients

Seventy-five patients of both genders who needed surgical treatment due to CRC confirmed by radiological, colonoscopic, and histological findings were included in the cross-sectional study conducted from June 2014 to January 2016 on Clinic for General and Abdominal Surgery, Clinical Center of the University of Sarajevo. The patient group consisted of 40 males and 35 females with mean age of 65.7 years (47-78 years). Patients who had had some other benign or malignant neoplasia, patients treated by radiotherapy or chemotherapy before surgery treatment, and those with inflammatory bowel disease or a known history of familial adenomatous polyposis were not included in the study. The control group of 20 healthy individuals was recruited from the people of the appropriate age and gender subjected to preventive screening at the Counseling Centre for Gastroenterology, who were without family history of cancer or clinical signs of malignant or inflammatory disease. All the subjects were included in the study with obtained informed consent. The study protocol was approved by the local Ethics committee. The study have been performed in accordance with the ethical standards laid down in the 1964 Declaration of Helsinki.

### 2.2. Material

Five mL of peripheral venous blood was sampled from the patients with colorectal cancer before their surgical treatment and from controls on the day of physical examination. The blood was collected in BD Vacutainer test tube with no additive and immediately transported to the laboratory. After spontaneous precipitation of the sample for 20 minutes, the same were centrifuged at 3000 rpm for 10 minutes, with separation of serum into three aliquots. Two serum samples were stored at -80°C until analysis of MMP-9 and IL-6 serum concentration, while CRP serum level was determined on the day of the blood sample. MMP-9 and IL-6 serum concentration was determined on duplicate aliquots of each sample using the technique of enzyme-linked immunosorbent assay (ELISA) according to the manufacturer's instructions (R&D Systems, Inc.; RD-DMP900). Reading of the results was carried out spectrophotometrically at 450 nm on a plate reader BioTek ELX50, with the correction wavelength at 540 nm or 570 nm. Measured MMP-9 serum concentration was expressed in nanograms per milliliter (ng/mL), while IL-6 concentration was expressed in picograms per milliliter (pg/mL). CRP serum level was estimated by turbidimetric immunoassay on the Dimension x Pand Plus system (Siemens) and expressed in milligram per liter (mg/L).

### 2.3. Methods

Colorectal cancer surgery was performed according to the principle of* en bloc* resection of colon cancer with associated lymph-vascular arcade. After resection and macroscopic examination of surgically obtained tissue material, samples of tumor were fixed in 10% phosphate buffered formalin. Paraffin blocks were cut to a thickness of 3-5 microns. The obtained sections were stained with standard hematoxylin and eosin stain, followed by determination of histological type and grade of colon cancer, the depth invasion of the tumor in the intestine wall (pT), and the number of regional metastatic lymph nodes (pN) [17]. Stage of colorectal cancer was determined according to the TNM classification of the American Association of Cancer (AJCC) in 2010, in which “T” marks the depth of invasion of the bowel wall, “N” the number of lymph nodes involved in metastatic process, and “M” means metastasis, while wider staging was defined by numbers from I to IV [18].

### 2.4. Statistical Analysis

The normality of data distribution was determined by Shapiro-Wilk test. All data were expressed as median and interquartile range. Mann–Whitney U test was used to compare the differences in parameters between the patient and control groups. Mood's Median test was used for statistical evaluation of three or more groups. Spearman's Rank correlation was used to assess the association between parameters in the patient group. The predictive significance of monitored biomarkers in assessing the stage of colorectal cancer was determined by multinomial logistic regression. The level of significance was set at* p* <0.05. Statistical analysis was performed by the Minitab 17 Software for Windows (Minitab, Inc. 2014).

## 3. Results

The most common localization of colon cancer was estimated in the rectum (24 patients, 32%) and then in the sigmoid colon (11 patients, 14.7%), descending colon (10 patients, 17.3%), while the rectosigmoid location of colon cancer was found in 9 (12%) patients. Right colon was affected in 17 (22.7%) patients, and transverse colon in 4 patients (5.3%). Dominant histological tumor type was adenocarcinoma (100%), predominantly grade 2 (65.5%).

Compared to controls, in the patient group there was a significant different increase in IL-6, CRP and MMP-9 serum levels according to the stage of CRC (Table 1). A significant increase in CRP serum value was associated with an increase in CRC stage (from 7.2 (2.3-13.0) mg/L in stage II to 38.6 (21.4-74.5) mg/L in stage IV,* p* <0.001), whereas MMP-9 serum level was significantly higher in stage III (458.4 (447.0-464.1) ng/mL) and stage IV (459.7 (453.2-469.6) ng/mL) compared to MMP-9 serum value in stage II CRC (434.8 (373.3-447.4) ng/mL) (*p* <0.001). IL-6 serum concentration was significantly higher in the stage IV (28.8 (10.4-75.6) pg/mL) compared to stage III (6.5 (0.2-21.3) pg/mL) and stage II CRC (2.5 (0.0-4.0) pg/mL) (*p* <0.001).

With regard to the depth invasion of the bowel wall (pT), CRP serum level was increased with increasing the depth of tumor invasion from pT2 to pT4 bowel wall infiltration (from 17.75 (7.3-20.25) mg/L to 29.7 (14.7-50) mg/L,* p* <0.05), while there were no important difference in IL-6 and MMP-9 serum levels between the patients with pT2-pT4.

However, IL-6, CRP, and MMP-9 serum concentrations were significantly higher in CRC patients with N1 lymph node status (1-3 regional metastatic lymph nodes) and N2 involvement of regional lymph nodes (4 or more metastatic lymph nodes) compared to patients with absence of invasion in regional lymph nodes (N0) (*p *<0.001).

**Figure 1:**
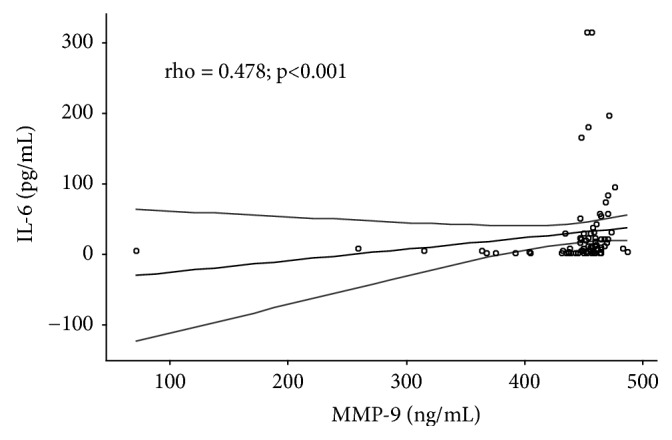
Correlation between IL-6 and MMP-9 serum levels in patients with colorectal cancer. rho = correlation coefficient; dashed line indicates 95% confidence interval (95% CI); stages II, III, and IV CRC have been taken into account for statistical analyses, a total of 75 patients.

**Figure 2:**
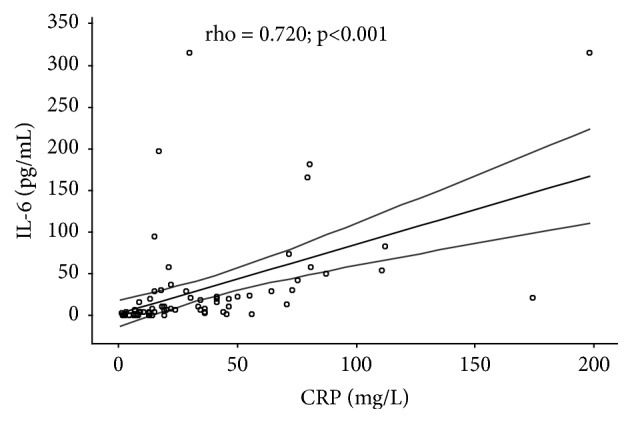
Correlation between IL-6 and CRP serum levels in patients with colorectal cancer. rho = correlation coefficient; dashed line indicates 95% confidence interval (95% CI); stages II, III, and IV CRC have been taken into account for statistical analyses, a total of 75 patients.

**Figure 3:**
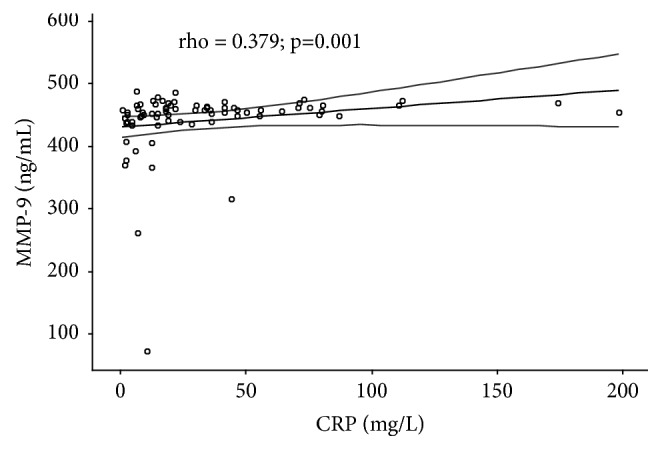
Correlation between MMP-9 and CRP serum levels in patients with colorectal cancer. rho = correlation coefficient; dashed line indicates 95% confidence interval (95% CI); stages II, III, and IV CRC have been taken into account for statistical analyses, a total of 75 patients.

By analyzing the correlation of the investigated parameters in all patients with CRC (stages II, III, and IV, a total of 75 patients), significant positive correlation between IL-6 and MMP-9 serum levels was confirmed (rho=0.478;* p* <0.001) (Figure 1), as well as between IL-6 and CRP serum levels (rho=0.720;* p* <0.001) (Figure 2) and between MMP-9 and CRP serum levels (rho = 0.379;* p *= 0.001) (Figure 3).

In the multinomial logical regression analysis of independent predictors of CRC staging, it has been shown that from II to IV stages of this cancer all tracked biomarkers, other than IL-6, have a significant predictive value (*p* <0.05) in assessing colorectal cancer stage, but from stages III to IV none of the monitored biomarkers had not a significant predictive value in the assess of colorectal cancer stage (Table 2).

## 4. Discussion

Chronic inflammatory bowel diseases, particularly ulcerative colitis, increase the risk of developing colorectal cancer, which emphasizes the importance of chronic inflammation in colon carcinogenesis. In addition, the cells of colorectal cancer, stimulated by preexisting inflammation or carried by intrinsic pathways related to genetic defects, may secrete a variety of cytokines and activated local stromal cells [19].

The role of immune cells and their products in progression and metastasis of colorectal cancer is not yet well known. Recently published studies suggested that, among cytokines involved in intestinal tumor genesis associated with inflammation, an important role belongs to IL-6 [20, 21]. This cytokine is considered a key link between inflammation and tumor genesis due to the confirmed association of IL-6 expression and CRC prognosis, as well as association with IBD. The mechanisms by which IL-6 contributes to the pathogenesis of IBD and cancer are not fully understood. The action of IL-6 is conditioned by binding to IL-6 receptor-alpha on target cells and then by binding this complex to the receptor subunit gp130 leading to the activation of the JAK/STAT signaling pathway in classic signaling [22]. Belluco with associates [23] described an association of a common functional single G>C base polymorphism in the human IL-6 gene promoter (chromosome 7p21) with serum levels of this cytokine in patients with CRC, particularly in the presence of hepatic metastasis.

In our research IL-6 serum levels were statistically significantly different between controls and patients with CRC and showed marked increase with increasing stages of colorectal cancer. Median IL-6 serum levels statistically significantly differed between the individual stages of CRC, reaching the highest level in stage IV. We found that IL-6 serum levels were higher in patients suffering from CRC with N1 and N2 lymph nodes status compared to patients without metastasis to regional lymph nodes (N0). Median IL-6 serum values increased with an increase of invasion in the intestinal wall (pT), but without statistical significance.

Several studies have found an increased expression of IL-6 in patients with CRC, where IL-6 levels are elevated in the serum of patients and in tumor tissue itself [10, 24]. The results of the recently published Chinese study suggest that IL-6 expression in the CRC tissue is associated with tumor TNM stage, invasion depth, and lymph node metastasis in CRC [25]. Finding a parallel increase in IL-6 serum levels with increasing stage of colorectal cancer and increasing metastatic regional lymph nodes in our study supports that opinion. Since intestinal epithelial cells usually do not express the membrane-bound IL-6R, some authors consider that effects of IL-6 on colorectal cancer cells are mainly modulated through IL-6 trans-signaling promoting tumor cell proliferation and inhibiting apoptosis through gp130 activation on tumor cells and then with signaling through Janus kinases (JAKs) and signal transducer and activator of transcription 3 (STAT3) [24, 26].

Inflammatory cytokines are potent matrix metalloproteinase (MMP) activators. The study of Kothari and associates [27] demonstrated that IL-6 induces macrophage expression of MMP-9 via Cox-2-dependent and Cox-2-independent mechanisms, which has been directly associated with the pathogenesis of chronic inflammatory diseases and cancer. MMP-9 serum levels were found to be elevated in many studies about CRC [15, 28, 29]. Our results indicate that MMP-9 serum concentration was significantly higher in patients with colorectal cancer than in the control group and significantly increased in stages III and IV CRC compared to stage II suggesting that MMP-9 is primarily involved in the occurrence of metastatic colorectal cancer. The similar relationship was confirmed according to the tumor size (pT) and the spread of tumor process to the regional lymph nodes (pN). A study by Biasi and associates [30] has indicated that MMP-9 serum levels significantly increased during the carcinogenic process in human colorectal tract, with a significant difference in the mean value of this biomarker in stage III CRC compared to control. That research confirmed a significant correlation between MMP-9 and IL-8 serum levels precisely in stage III CRC, while our study confirmed a significant positive correlation between MMP-9 and IL-6 serum levels, as well as between MMP-9 and CRP serum levels in all patients with CRC indicating the mutual stimulated interaction of these biomarkers in colorectal carcinogenesis. A study of Kothari et al. [27] demonstrated that IL-6 induces macrophage expression of MMP-9, which has been directly associated with the pathogenesis of chronic inflammatory diseases and cancer. Inflammatory cytokines are believed to increase matrix metalloproteinase (MMP) dysfunction, while MMPs increase tissue inflammation.

We have found that CRP serum level in patients with CRC was significantly higher with increasing stage of this disease, as well as with increasing of tumor bowel wall infiltration and regional lymph nodes invasion, reaching the highest values in the IV stage CRC. Significant positive correlation between IL-6 and CRP serum levels was confirmed in the patients with CRC strongly suggesting that CRP as a general marker of inflammation should be combined with the other inflammatory molecules in patients with colorectal cancer. Italian authors also demonstrated an increase in CRP serum levels in the patients with colorectal cancer, particularly in the second and third stages of the disease [30], comparable to the tendency we found.

Several meta-analyses and systematic reviews have assessed the association between IL-6 serum levels and risk of CRC, whereas these pooled results were insignificant [31, 32]. Kakourou with associates [31] reported a significantly positive association between IL-6 serum level and risk of colon cancer, but the results were opposite for rectal cancer. The results of our study indicate that all tracked biomarkers, other than IL-6, have a significant predictive value in the assessment of stage II to stage IV CRC but not from stage III to stage IV which suggests the prognostic importance of the stage II in colon carcinogenesis.

This is one of the few studies which have followed simultaneously several biomarkers in patients with CRC in order to identify an inflammatory network that contributes to the progression of CRC. This study has a few limitations. This is a study of a single center, which is the reason for involving a relatively small number of surgically treated patients with CRC. A comparison between rectal cancer and other parts of the colon as well as the analysis the following biomarkers after surgical removal of colon cancer had not been made.

## 5. Conclusion

IL-6, CRP, and MMP-9 serum levels showed very similar trends, increasing concomitantly and reaching the highest values in stage IV CRC, indicating they are involved in tumor promotion and proliferation. Our results showed that there is strong association between MMP-9 and CRP serum levels and progression of colorectal cancer. MMP-9 and CRP serum levels have a significant predictive value in the assessment stage II to IV colorectal cancer which points to persisting inflammation-related process and its importance in the colorectal tumor genesis. These biomarkers in combination might be considered as a potential tool for monitoring colorectal cancer progression.

## Figures and Tables

**Table 1:** IL-6, CRP, and MMP-9 serum concentrations in relation to clinicopathological features of the colorectal cancer.

		CRC stage			
Variabe	Control (n=20)	II (n=22)	III (n=27)	IV (n=26)	*p* Value

IL-6 (pg/mL)	0.0 (0.0-0.0)^a^	2.5 (0.0-4.0)^b^	6.5 (0.2-21.3)^b^	28.8 (10.4-75.6)^c^	<0.001
CRP (mg/L)	2.3 (0.7-3.8)^a^	7.2 (2.3-13.0)^b^	19.0 (8.5-45.0)^c^	38.6 (21.4-74.5)^d^	<0.001
MMP-9 (ng/mL)	310.6 (205.0-356.3)^a^	434.8 (373.3-447.4)^b^	458.4 (447.0-464.1)^c^	459.7 (453.2-469.6)^c^	<0.001

		Bowel wall infiltration (pT)			
		pT2 (n=10)	pT3 (n=38)	pT4 (n=27)	*p* Value

IL-6 (pg/mL)	0.0 (0.0-0.0)^a^	6.6 (0.3-21.1)^b^	8.9 (2.1-29.2)^b^	6.5 (0.2-21.3)^b^	0.306
CRP (mg/L)	2.3 (0.7-3.8)^a^	17.75 (7.3-20.25)^b^	19.9 (7.9-45.7)^b^	29.7 (14.7-50)^c^	<0.05
MMP-9 (ng/mL)	310.6 (205.0-356.3)^a^	450.35 (438-454.8)^b^	450.9 (435.8-460.5)^b^	458.4 (447-464.1)^b^	0.103

		Lymph nodes invasion (pN)			
		pN0 (n=25)	pN1 (n=26)	pN2 (n=24)	*p* Value

IL-6 (pg/mL)	0.0 (0.0-0.0)^a^	3.0 (0.0-5.2)^b^	10.3 (2.5-44.8)^c^	20.1 (6.4-46.7)^c^	<0.001
CRP (mg/L)	2.3 (0.7-3.8)^a^	8.3 (2.4-20.3)^b^	26.7 (15.9-59.3)^c^	33.7 (14.7-68.5)^c^	<0.001
MMP-9 (ng/mL)	310.6 (205.0-356.3)^a^	437.4 (383.3-448.8)^b^	458.9 (452.8-465.4)^c^	458.2 (449.8-464.6)^c^	<0.001

Data are presented as median and interquartile range q1-q3 (25%-75%).

^abcd^Values in the same row that do not contain a common lettering differ significantly on the level *p* <0.05.

CRC: colorectal cancer; IL-6: interleukin-6; CRP: C-reactive protein; MMP-9: matrix metalloproteinase-9; pT2: tumor invades muscularis propria; pT3: the tumor invades through the muscularis propria in the pericolic tissue; pT4: the tumor penetrates the surface of visceral peritoneum or other organs; pN0: no invasion to regional lymph nodes; pN1: 1-3 regional metastatic lymph nodes; pN2: 4 or more metastatic lymph nodes.

**Table 2:** Independent predictors of the colorectal cancer stage.

					95% CI	
Predictor	coefficient	SE	*p* Value	OR	lower	upper

II/IV stage of CRC						

Constant	108.075	44.860	0.016			
MMP-9 (ng/mL)	-0.208	0.091	0.022	0.81	0.68	0.97
CRP (mg/L)	-0.216	0.103	0.036	0.81	0.66	0.99
IL-6 (pg/mL)	0.016	0.017	0.360	1.02	0.98	1.05

III/IV stage of CRC						

Constant	21.663	14.244	0.128			
MMP-9 (ng/mL)	-0.042	0.030	0.164	0.96	0.90	1.02
CRP (mg/L)	-0.022	0.013	0.082	0.98	0.95	1.00
IL-6 (pg/mL)	-0.011	0.008	0.182	0.99	0.97	1.01

CRC: colorectal cancer; SE: standard error of coefficient; OR: odds ratio; CI: confidence interval.

## Data Availability

The data used to support the findings of this study are available from the corresponding author upon request.
